# Ulceration of the Colon in the Neighborhood of Gunshot Wounds

**Published:** 1917-11

**Authors:** 


**Ulceration of the Colon in the Neighborhood of Gunshot Wounds.** By John Shaw DUNN and Hamilton DRUMOND. Abstracted from the *British Journal of Surgery*, July, 1917.

Wounds of the colon by missiles perforating the viscus seem to differ only slightly from lesions produced in the small intestine. If quickly detected and closed, they may be expected to heal readily.

The lesions treated in this paper, however, differ markedly from such direct wounds both in causation and prognosis. When a missile passes near but does not touch the wall of the colon, extensive ulceration of the colic mucous membrane seems to ensue, and is likely to extend deeply into the intestinal wall, resulting in perforation and peritonitis or retroperitoneal cellulitis : hence the gravity of this injury apart from other lesions accompanying it. Such ulceration, however, is sometimes present in cases which recover. Bowly testifies that fecal fistulae often develop in relation to wounds that do not penetrate the intestine.

In notes on six cases the author establishes the fact that extensive ulceration of the mucous membrane occurred when the external aspect of the bowel indicated no such condition, and when the missile had not perforated the colon, or even come into direct contact with its outer wall.

The lesions were fully developed at a remarkably early period — from 7 to 26 hours — after the injury. They may be explained by the following facts :

There was always some laceration of the muscular coats, though this was usually slight compared to the lesion in the mucosa, and was not accompanied by loss of muscular substance. In one case damage to the muscular layers was clearly produced by a dragging effect through strands of fibrous tissues or blood-vessels.

In other cases the effects were probably due to sudden impact on a gas-filled sac, resulting in bursting at weak points, the lacerations occurring most commonly in the unsupported sacculi. It is to be noted also that lesions in the muscular coats have been found only in fixed portions of the colon. Another fact of interest was that many of the perforating vessels to the mucosa in the damaged areas were torn across at the points where they passed from the muscular to the sub-mucous coats, accounting for the great loss of mucous membrane where the injury of the muscle was comparatively trivial. In reality the patch of mucosa had been killed by loss of blood supply. The content of the bowel, no doubt, caused early infection in the damaged tissues.

Adequate drainage is of utmost importance in all cases of visible bruising of the colon walls, especially in the fixed ascending and descending parts.

